# Seroclearance of hepatitis B surface antigen following hepatitis E exacerbation on chronic hepatitis E and B dual infection in a renal transplant recipient: a case report

**DOI:** 10.1186/s13256-018-1586-2

**Published:** 2018-02-28

**Authors:** Chau-Ting Yeh, Christopher Sung-Huan Yeh, Yu-De Chu, Yang-Jen Chiang

**Affiliations:** 1 Liver Research Center, Chang Gung Memorial Hospital, 5, Fu-Shin Street, Kuei-Shan District, Taoyuan, Taiwan; 2 Department of Cognitive Science, College of Letters and Science, University of California Los Angeles, Los Angeles, USA; 3 Department of Urology and Renal Transplantation, Chang Gung Memorial Hospital, Taoyuan, Taiwan

**Keywords:** Hepatitis B, Hepatitis E, Coinfection

## Abstract

**Background:**

Hepatitis E virus infection usually causes an acute and self-resolving hepatitis. In areas where chronic hepatitis B virus infection is prevalent, acute hepatitis E virus superinfection on chronic hepatitis B virus infection occurs sporadically. In recent years, however, chronic hepatitis E virus infection has been recognized in patients under immunosuppressant therapy. To the best of our knowledge, cases involving patients with chronic hepatitis E virus and hepatitis B virus dual infection have never been reported.

**Case presentation:**

A 47-year-old Taiwanese woman who was a renal transplant recipient with chronic hepatitis B virus infection was under immunosuppressant and antiviral treatment. An episode of hepatitis B exacerbation developed due to withdrawal of antiviral treatment against advice, but the flare subsided following antiviral re-treatments. However, an episode of hepatitis exacerbation developed following removal of the renal graft because of graft failure. During the hepatitis flare, she was still under successful antiviral suppression against hepatitis B virus, while her serum samples were positive for hepatitis E virus RNA. Following the hepatitis flare, seroclearance of hepatitis B virus surface antigen developed. From then on, she was under regular hemodialysis. Five years later, another episode of mild hepatitis exacerbation occurred again with positive serum hepatitis E virus RNA. Tracing back the longitudinal serum samples, serum hepatitis E virus RNA was persistently positive throughout the course. This patient was thus recognized to have chronic hepatitis E virus and hepatitis B virus dual infection with intermittent hepatitis E exacerbations.

**Conclusions:**

In areas where chronic hepatitis B virus infection is prevalent, chronic hepatitis E virus coinfection can occur in organ transplant recipients receiving immunosuppressant. Intermittent hepatitis E exacerbations may develop, interfering with the status of hepatitis B virus infection.

## Background

Chronic hepatitis E virus (HEV) infection has been reported in immunocompromised patients [1]. In Asia, where hepatitis B virus (HBV) infection is highly prevalent, acute HEV superinfection on chronic HBV infection could occur sporadically. However, patients with dual chronic HEV and HBV infection have never been documented. Here, we reported a renal transplant recipient, who had dual chronic HEV and HBV infection. Interestingly, following an episode of acute hepatitis E exacerbation, seroclearance of HBV surface antigen (HBsAg) developed and our patient achieved a “functional cure” for chronic HBV infection.

## Case presentation

**Fig. 1:**
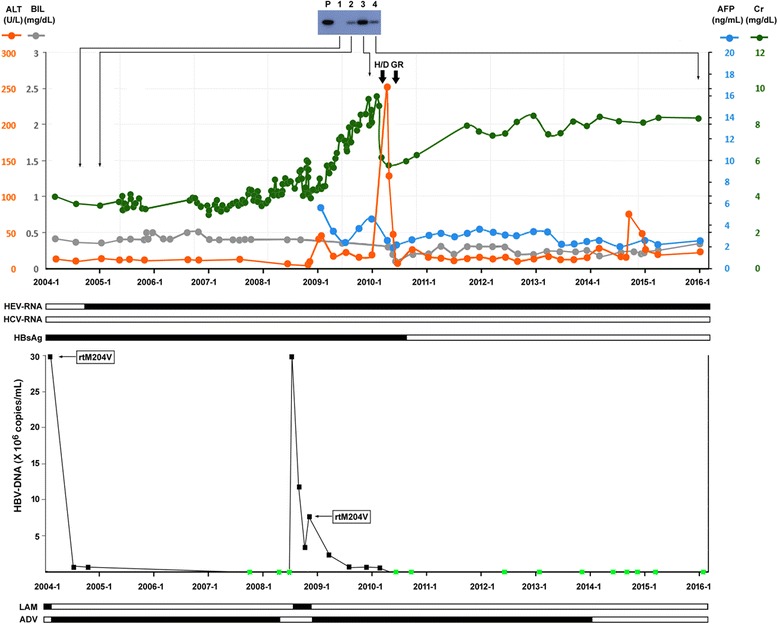
Clinical course of our patient with dual chronic hepatitis E and B infection. **Upper panel** Hepatitis E virus RNA was detected by reverse transcription-polymerase chain reaction followed by southern Blot analysis (*top*). *Lanes 1–4*, hepatitis E virus RNA detected from serum samples obtained from various time-points (*arrows*). Changes of alanine transaminase (*orange circles and line*), bilirubin (*grey*), alpha-fetoprotein (*blue*), and creatinine (*green*) along the course are depicted. Positive (*solid horizontal bar*) status of hepatitis E virus RNA; hepatitis C virus RNA and hepatitis B virus surface antigen are shown. **Lower panel** Changes of hepatitis B virus DNA along the course. *Solid squares*, positive hepatitis B virus DNA; *green squares*, hepatitis B virus DNA undetectable. Time-points to detect the rtM204V mutation are marked by *arrows*. *Bottom*, the periods of time when lamivudine or adefovir were given (*solid horizontal bars*). All serological and molecular virology assays are described in our previous publications [6, 7]. *ADV* adefovir, *AFP* alpha-fetoprotein, *ALT* alanine transaminase, *BIL* bilirubin, *Cr* creatinine, *GR* graft removed, *HBsAg* hepatitis B virus surface antigen, *HBV* hepatitis B virus, *HCV* hepatitis C virus, *H/D* hemodialysis, *HEV* hepatitis E virus, *LAM* lamivudine, *P* positive hybridization control

A 47-year-old Taiwanese woman, a housewife, who was an HBV carrier, had received renal transplantation in 2002 and was treated with an immunosuppressant. Lamivudine was provided to maintain virological suppression for HBV. She was referred to the Liver Clinic of Chang Gung Memorial Hospital in January 2004 due to elevated HBV DNA levels (31.2 × 10^6^ copies/mL). She denied major systemic diseases other than chronic hepatitis B and renal disease. No documented hereditary diseases or psychological disorders in her family were noted. She had an average socioeconomic status. On physical examination, she appeared normal with no icteric sclera, no hepatosplenomegaly, no spider nevi, and no caput medusae. She was positive for HBsAg, negative for HBV e antigen (HBeAg), and positive for anti-HBe antibody. No clinical evidence of liver cirrhosis was noted. Genotypic analysis revealed genotype B, precore stop codon G1896A mutation (+), basal core promoter mutation (−), and rtM204V/rtL180M mutation (+). She was switched to adefovir monotherapy and HBV DNA declined rapidly (Fig. 1, lower panel). HBV DNA became undetectable in August 2007. She stopped antiviral treatment in February 2008 against advice. However, relapse of viremia (29.3 × 10^6^ copies/mL) developed and she was treated with lamivudine again for no detectable genotypic resistance (insurance coverage policy), but switched to adefovir shortly afterwards because of emergence of rtM204V/rtL180M mutants accompanied by a mild hepatitis flare: alanine transaminase (ALT) 49 U/L. From then on, maintained suppression of HBV DNA was achieved. HBV DNA was undetectable since March 2010. The flares in 2004 and late 2008 were considered flares of hepatitis B because of the accompanied elevations of HBV DNA levels with normalization of ALT after suppression of HBV DNA.

Our patient suffered from graft rejection after renal transplantation and was treated with immunosuppressant drugs, including tacrolimus (3 mg/day), mycophenolate mofetil (500 mg twice a day), and prednisolone (10 mg/day). Her creatinine levels were between 3 and 4 mg/dL initially, but gradually elevated to 10 mg/dL despite increasing dosage of immunosuppressants. In January 2010, obstruction of graft ureter was found and hemodialysis resumed. Immunosuppressants were withdrawn and an episode of hepatitis flare occurred with ALT elevation to 251 U/L. After normalization of ALT, the renal graft was removed by operation. No HBV DNA elevation was detected before and during the flare. Throughout the flare, HBV DNA was suppressed to less than 10^4^ copies/mL by adefovir. No known hepatotoxic drug was in use during this period. Anti-hepatitis C virus (HCV) antibody was positive since January 2008 but HCV RNA was negative throughout the clinical course. IgM anti-hepatitis A virus antibody, anti-hepatitis D virus (HDV) antibody, and HDV RNA were all tested negative. Subsequently, HEV RNA was found to be positive by reverse transcription-polymerase chain reaction (RT-PCR) and Southern blot analysis (Fig. 1, upper panel). Sequence analysis revealed genotype 3. Positive HEV RNA could be traced back to January 2005 but before that it was negative. Strikingly, seroclearance of HBsAg occurred in July 2010. Before this flare, the quantitative level of HBsAg was 25,000 IU/mL. It remained negative thereafter and adefovir was stopped in January 2014 with no subsequent virological relapse. Another episode of ALT elevation (to 73 U/L) was found during October to November 2014 with no positive hepatitis A to D markers. HEV RNA continued to be positive (final check was performed in January 2016). IgG anti-HEV was positive. Ribavirin was not given because of the low hemoglobin level in this patient who received hemodialysis. Anti-HBs antibody was negative throughout the course.

## Discussion

Chronic HBV infection is highly prevalent in Asia. Therefore, acute but self-resolving HEV superinfection on chronic HBV infection can occur sporadically [2, 3]. Because chronic HEV infection can develop in patients under immunosuppressants, dual chronic HEV and HBV infection should be found in patients receiving organ transplantation, especially in an HBV endemic area. Here we identified a renal transplant recipient with chronic dual HEV and HBV infection. In this reported case of a patient, the dual chronic hepatitis infection was mostly asymptomatic during the clinical course. At that time, HBV infection was controlled by either lamivudine or adefovir monotherapy (if resistance developed), but not by add-on therapy because of high cost of adefovir. In this patient, HBV DNA could be suppressed to a low or undetectable level. The actual cause of hepatitis exacerbation preceded by HEV RNA elevation in January 2010 was unknown. A previous study showed that calcineurin inhibitors promoted HEV replication but mycophenolic acid inhibited HEV replication [4]. A disproportionate increase of the two immunosuppressants during graft rejection could result in fluctuating levels of HEV replication. On the other hand, withdrawal of immunosuppressant agents could increase host immune responses, and partly contribute to the exacerbation. No other causes of hepatitis could be identified other than HEV-related hepatitis flare. It is inspiring to find that HBsAg seroclearance could develop following a HEV-related hepatitis flare. Current clinical trials for new anti-HBV therapies are following a strategy similar to this scenario, where patients were under antiviral treatment to suppress HBV DNA and a novel immune-related agent or a new strategy to perturb immune responses was applied to induce seroclearance of HBsAg. Seroclearance of HBsAg was considered a “functional cure” for HBV infection [5]. It is worth noting that HEV-induced immune response can result in HBsAg clearance.

A second episode of mild hepatitis E exacerbation developed 5 years after the removal of the renal graft and withdrawal of immunosuppressant. Our patient was under regular hemodialysis at that time and remained persistently positive for serum HEV RNA.

## Conclusions

This case report revealed that chronic dual HEV and HBV infection could occur in areas where hepatitis B is prevalent. Hepatitis exacerbations of HEV were noted, resulting in seroclearance of HBsAg. Serum HEV RNA persisted even after immunosuppressant was stopped, while our patient was under hemodialysis.

## Abbreviations

ALT: Alanine transaminase
HBeAg: Hepatitis B virus e antigen
HBsAg: Hepatitis B virus surface antigen
HBV: Hepatitis B virus
HCV: Hepatitis C virus
HDV: Hepatitis D virus
HEV: Hepatitis E virus
RT-PCR: Reverse transcription-polymerase chain reaction
